# A Minority Within a Minority: Lived Meanings of Cancer Among Arab Young Adults in East Jerusalem

**DOI:** 10.1002/pon.70547

**Published:** 2026-07-14

**Authors:** Rola Abd Elgani, Yaffa Naomi Stokar

**Affiliations:** ^1^ Paul Baerwald School of Social Work and Social Welfare Hebrew University of Jerusalem Jerusalem Israel; ^2^ Sharett Institute of Oncology Hadassah Medical Center Jerusalem Israel

**Keywords:** arabs, cultural competency, disclosure, minority groups, psycho‐oncology, qualitative research, social stigma, socioeconomic disparities in health, trust, young adult

## Abstract

**Objective:**

Young adulthood is a socially and biographically formative life stage, yet young adults from ethnic‐national minority groups remain underrepresented in psycho‐oncology research. This study examined how Arab young adults (YA) from East Jerusalem make meaning of cancer while receiving treatment in predominantly Jewish‐Israeli hospitals.

**Methods:**

Guided by a context‐informed, constructivist qualitative framework, we conducted semi‐structured, in‐depth interviews with 15 Arab YA (aged 23–45) who were undergoing active cancer treatment. Interviews were conducted in Arabic, transcribed verbatim, and analyzed using reflexive thematic analysis to capture culturally and developmentally embedded meanings of illness and care.

**Results:**

Two interrelated sets of lived meanings emerged. First, participants described cancer through culturally embedded frameworks shaped by stigma, secrecy, family responsibility, and faith‐based interpretations that provided meaning and emotional containment. These meanings were closely tied to the future‐oriented concerns and relational obligations of young adulthood. Second, participants experienced Israeli hospitals as ambivalent institutional spaces: often perceived as safe, compassionate, and politically neutral yet at times marked by cultural misattunement, language barriers, and subtle inequality. Together, the findings show that cancer was experienced not only as a medical condition but as a deeply contextualized social and developmental experience.

**Conclusions:**

The experience of cancer among Arab YA from East Jerusalem is shaped by the intersection of developmental stage, minority positioning, and institutional context. These findings highlight the importance of context‐informed and culturally responsive psycho‐oncology care for young patients from socially and politically marginalized backgrounds.

## 1 Introduction

### 1.1 Cancer Among Arab Young Adults in Israel

Cancer in young adulthood disrupts not only the body but also the social and developmental trajectories that define this life stage, including education, work, independence, intimacy, and family life. For Arab young adults (YA) in Israel, these disruptions unfold within layered social, cultural, and political contexts that shape how illness is understood, concealed, and endured. Young adulthood is increasingly recognized as a distinct developmental period with unique psychosocial vulnerabilities, yet the experiences of young adults from ethnic‐national minority groups remain insufficiently theorized.

Although cancer in young adults is relatively rare, its psychosocial impact is profound. In Israel, approximately seven young adults aged 20–44 are diagnosed with cancer each day, with about one in ten cases occurring among individuals from the Israeli Arab community [1]. Despite Israel's ethnic and religious diversity—Jews and other groups comprise 79% of the population, and Muslims, predominantly Arabs, 21% [2]—research on the psychosocial dimensions of cancer among Arab patients remains scarce. Existing studies have addressed cultural perceptions of cancer within the Arab population broadly [3, 4] but have not examined the distinct experiences of Arab YA navigating cancer at the intersection of culture, age, and minority status. This gap is particularly notable given the increasing incidence of cancer among Arab YA in recent years [1]. Although cancer care in Israeli hospitals may also intersect with the broader Palestinian context, the present study focuses specifically on Arab young adults from East Jerusalem, whose experiences are shaped by a distinct legal, linguistic, institutional, and sociopolitical context.

### 1.2 Cancer During Young Adulthood

Although young adulthood is often used in oncology as a single descriptive category, it does not represent a uniform developmental stage. In cancer care and research, adolescents and young adults are commonly defined broadly as ages 15–39, while some national and clinical frameworks extend the upper boundary into the mid‐40s, particularly when addressing service provision and psychosocial care needs. This is the case in Israel, where designated clinics in all major medical centers and additional services for YA with cancer, serve people aged 18–44 [5, 6].

This age range should be understood as comprising multiple phases of adulthood rather than one cohesive psychosocial group, as individuals may differ markedly in their position with respect to identity consolidation, autonomy, romantic partnership, educational and occupational trajectories, parenthood, and long‐term life planning [7, 8, 9, 10]. Thus, while the term young adults remains clinically useful, it may obscure meaningful developmental variation between those in earlier adulthood, who are often navigating emerging independence, educational or vocational entry, and the formation of intimate relationships, and those in later young adulthood, who are more likely to be managing long‐term partnership, parenthood, financial responsibility, and established adult roles [7, 8, 11].

Viewing young adulthood as developmentally heterogeneous also clarifies why cancer may carry different meanings and consequences across this age span. Although many challenges are shared, the illness is experienced within distinct developmental, relational, and social contexts that shape both psychosocial burden and coping [12]. Cancer interrupts this progression, forcing patients to confront unique challenges, including early confrontation with mortality, increased dependence on caregivers, altered physical appearance, infertility, and disruptions to social and professional life [13]. Recent qualitative work demonstrates that adolescent and young adult cancer patients not only experience intense emotional disruptions across multiple domains but also encounter substantial barriers to accessing psychosocial support, often shaped by stigma and avoidance mechanisms [14].

For minorities and marginalized groups, such as Arab YA in Israel, illness also interacts with systemic inequities—economic, linguistic, and political—that amplify vulnerability [15, 16]. From a psychological perspective, these are not only emotional but existential, affecting well‐being, treatment adherence, and help‐seeking behavior. Yet, culturally contextualized research on these phenomena remains scarce.

### 1.3 Cancer in the Arab Society in Israel

Arab society in Israel is heterogeneous—comprising Muslims, Christians, and Druze—but united by shared values emphasizing family cohesion, modesty, and collectivism [17, 18]. Religious observance varies from secular to orthodox. Within this framework, illness carries both spiritual and moral meanings shaping ways Arab individuals perceive and respond to illness [19]. Cancer is often viewed as divinely ordained, a test or punishment that calls for patience and faith [20]. While such beliefs may offer solace, they can also lead to distress and social isolation.

A study on Israeli Arab women's attitudes toward breast and cervical cancer found that some viewed the disease as “a punishment or a test devised by God” [20]. Similarly, a comparative study of Israeli Jewish and Arab women reported lower screening adherence and survival rates among Arab women. Cultural barriers, such as social stigma, fear of gossip, lack of confidentiality and support, and religious conflicts between accepting fate and proactive health management, contributed to these disparities [21]. Simultaneously, structural inequalities, including poverty, discrimination, and unequal access to healthcare further compound the psychosocial strain of illness [19, 22].

### 1.4 The Case of East Jerusalem: A Minority Within a Minority

East Jerusalem epitomizes the complexity of identity and belonging for Arab residents. Approximately 367,000 Arabs live in Jerusalem, most in the eastern part of the city [23]. Since 1967, East Jerusalem has undergone profound political, legal, and administrative transformation. Following the June 1967 war, Israel occupied East Jerusalem, expanded Jerusalem's municipal boundaries, and incorporated additional territory into the city's municipal jurisdiction; however, this annexation has not been internationally recognized, and East Jerusalem continues to be regarded in United Nations and International Court of Justice frameworks as part of the occupied Palestinian territory [24, 25]. Under Israeli domestic law, however, Jerusalem is governed through Israeli municipal and state institutions [26]. In Palestinian statistical and administrative usage, the Jerusalem Governorate is divided into two subareas: J1, referring to the part of Jerusalem annexed by Israel in 1967 and included within the Israeli‐defined municipal boundary, and J2, referring to the remainder of the Jerusalem Governorate outside that boundary (Palestinian Central Bureau of Statistics) [27, 28]. This distinction reflects not only geography but also markedly different patterns of governance and daily life. Residents of J1 are more directly incorporated into Israeli municipal, taxation, labor, and health systems, whereas J2 remains more closely linked to the rest of the West Bank and Palestinian institutional structures [29, 30]. Consistent with this divide, PCBS reported that in 2013, 95.5% of residents in J1 used Israeli health insurance and 80.3% of households in J1 reported paying municipal tax, underscoring that J1 and J2 represent distinct legal and socio‐spatial realities within the broader Jerusalem Governorate [30].

Approximately 90% of the Palestinian population of East Jerusalem (J1) hold Israeli permanent‐resident status, whereas about 9% hold full Israeli citizenship [31]. Residents of J1 are entitled to Israeli municipal services, national health insurance, education, and social services, while their political rights and national representation remain limited [18, 32, 33]. The school system in East Jerusalem is divided among frameworks using the Israeli curriculum, the Palestinian curriculum, and mixed curricular models [34]. Although J1 residents are formally included in Jerusalem's municipal systems and can move throughout the city, substantial gaps in educational infrastructure and municipal services persist in East Jerusalem [34].

### 1.5 Navigating Cancer as a Minority Within a Minority

Against this background, the experience of cancer among East Jerusalem residents must be understood as more than a biomedical event. Rather, it unfolds within a broader social and institutional context that shapes access to care, communication, disclosure, and psychosocial coping. Unequal educational opportunities are also reflected in uneven opportunities for Hebrew acquisition and use in daily institutional life. As a result, Hebrew proficiency is often limited, creating significant barriers for cancer patients seeking medical care in Jewish‐Israeli hospitals and exacerbating communication difficulties, mistrust, and challenges in navigating care [18]. Communication with clinicians may also at times be mediated through family members, a pattern that can provide practical and emotional support but may also complicate privacy, disclosure, and patient autonomy in cancer care [35, 36]. Even when care is formally accessible, differences in language, communication style, and assumptions regarding disclosure may further affect trust and the therapeutic relationship in cross‐cultural clinical encounters [36, 37].

For many East Jerusalem residents, cancer carries layers of stigma, linked to hereditary fears, loss of marriage prospects, and social disgrace. The illness thus exists not only as a biological condition but as a socially mediated silence, one that reflects broader tensions between collectivist norms, emphasizing quiet suffering and concealing vulnerability, and personal coping, especially the possibility of seeking external support. Within this silence, individuals are expected to maintain composure and dignity, avoiding visible vulnerability to protect family honor [18]. Such stigma and secrecy may also shape when and how patients seek support, as selective disclosure can delay help‐seeking and limit access to psychosocial resources beyond the family [18, 38]. These dynamics render psychosocial support and self‐disclosure particularly complex. Taken together, the literature reviewed here suggests that cancer among Arab young adults in East Jerusalem is best understood as embedded in multiple intersecting contexts rather than through a single explanatory frame. In the present study, we therefore examine participants' accounts as shaped by sociocultural, developmental, institutional, and political realities, as these are mediated through family life, language, and religion.

### 1.6 The Current Study

Despite increasing awareness of cancer among YA and among Arab patients in Israel, to date no study has focused specifically on young adults from East Jerusalem, a population living at the intersection of national, cultural, and developmental boundaries. This study seeks to illuminate how Arab YA from East Jerusalem experience and make sense of cancer within their sociocultural and political context and how they navigate care within predominantly Jewish‐Israeli medical institutions. By privileging participants' narratives and examining meaning‐making processes through a context‐informed, constructivist lens, this study contributes to a more nuanced understanding of how culture, identity, and social structure can shape the lived experience of illness. In doing so, the analysis considers how illness meaning‐making is shaped across several intersecting domains, including sociocultural norms, developmental position within young adulthood, institutional encounters in healthcare, and the broader national context in which care is received. In this way, the study offers a contextually grounded account of illness meaning‐making in a population that remains largely absent from existing psycho‐oncology literature.

## 2 Methods

### 2.1 The Context‐Informed Framework

This study adopted a *context‐informed, constructivist qualitative approach* [39] to explore how Arab YA make sense of living with cancer within the sociopolitical, cultural, and institutional contexts of Israeli hospitals. Context is conceptualized not as background but as a dynamic, relational web of meanings—historical, social, and linguistic—through which experiences of illness are interpreted [40]. Accordingly, the analysis does not treat these as separate explanatory domains, but as overlapping contexts through which participants made sense of illness and care.

Within this perspective, meaning is co‐constructed between researchers and participants, shaped by cultural narratives, professional discourses, and power relations embedded in clinical encounters. This framework aligns with principles of clinical health psychology, emphasizing the inseparability of psychological adaptation and the cultural‐political environments in which people live [18].

### 2.2 Research Participants and Recruitment

Fifteen Arab YA (aged 23–45) participated in the study (see Table 1). All participants, except for one who chose not to disclose the exact location of residence within Jerusalem, resided in East Jerusalem and were undergoing active cancer treatment at Hadassah University Hospital‐Mt. Scopus and Hadassah University Hospital‐Ein Kerem, two tertiary hospitals in Jerusalem that employ and serve diverse Jewish and Arab populations.

**TABLE 1:** Participant characteristics.

#	Pseudo name	Age	Cancer type	Status in east Jerusalem	Level of education	Family status
1	Dima	38	Breast	East Jerusalem residency (J1)	Highschool graduate^c^	Married+3
2	Hanan	34	Breast	East Jerusalem residency (J1)	M.A.	Married+0
3	Heba	45	Metastatic colon	East Jerusalem residency (J1)	8 years	Married+5
4	Izz	27	Lymphoma	East Jerusalem residency (J1)	10 years	Married+2
5	Julia	34	Breast	Temporary East Jerusalem status^a^ (J1)	M.A.	Separated+3
6	Conan	23	Testicular	East Jerusalem residency (J1)	B.A.	Single+0
7	Maha	43	Breast	East Jerusalem residency (J1)	Highschool graduate^c^	Married+3
8	Mariam	32	Breast	East Jerusalem residency (J1)	B.A.	Married+2
9	Muhamad	38	Brain	East Jerusalem residency (J1)	14 years	Married+3
10	Naya	38	Breast	Israeli citizen (unknown^b^)	B.A.	Married+4
11	Rahma	45	Breast	East Jerusalem residency (J1)	Highschool graduate^c^	Married+5
12	Rana	36	Brain (GBM)	East Jerusalem residency (J1)	B.A.	Married+3
13	Reem	38	Breast	East Jerusalem residency (J2)	10 years	Married+5
14	Sara	34	Lymphoma	East Jerusalem residency (J1)	14 years	Married+2
15	Zina	40	Breast	East Jerusalem residency (J1)	17 years	Married+1

*Note:* J1‐referring to the part of east Jerusalem annexed by Israel in 1967 and included within the Israeli‐defined municipal boundary, recognized internationally as occupied Palestinian territory. J2‐the part of east Jerusalem not annexed by Israel, linked to the rest of the West Bank and Palestinian institutional structures, and mostly governed by the Palestinian Authority.

^a^ Temporary East Jerusalem status‐participant born abroad therefore not eligible for permanent residency status.

^b^ Unknown—the participant chose not to disclose their exact place of residence within Jerusalem for reasons of trust and confidentiality.

^c^ Highschool graduate—12 years of education.

Recruitment followed a combination of purposive and convenience sampling [41]. Eligible participants were Arab patients aged 18–44 who were physically and emotionally able to engage in a one‐time, face‐to‐face interview during treatment. Treating oncologists introduced the study to eligible patients and, upon permission, referred them to the research team. However, due to recruitment challenges related to trust, secrecy, and concerns about confidentiality, as well as fluctuations in some participants' physical health, there was at times a delay between the initial approach and the actual interview. As a result, two participants who met eligibility criteria when first approached were 45 years old by the time the interviews took place.

The first author, an Arab clinical social work graduate student and native Arabic speaker, contacted potential participants, provided detailed explanations in Arabic, and ensured voluntary participation. Building trust was essential. Several patients initially declined participation, citing concerns about privacy and community exposure, concerns that later emerged as a significant theme in the analysis. Data collection continued until information redundancy was reached, when subsequent interviews no longer produced new conceptual insights. This iterative process prioritized depth and cultural sensitivity over numerical saturation.

### 2.3 Research Procedure

Semi‐structured, in‐depth interviews were conducted between April 2022 and January 2023. In keeping with the context‐informed lens, interviews were approached as collaborative meaning‐making encounters rather than data extraction [42].

All interviews were conducted in Arabic by the first author, following written informed consent, in quiet spaces within oncology units, balancing participants' comfort with confidentiality. Interviews lasted 60–90 min and followed an open‐ended guide beginning with: *“We are interested in understanding the psychological and social experiences of young Arabs with cancer between the ages of 18–45. First, I would like to hear about your life story in the context of the illness and how you have experienced life since your diagnosis.”* This opening allowed participants to share their experiences freely. Subsequent prompts explored relationships, coping, faith, stigma, and sources of resilience. The conversational flow was flexible, allowing participants to lead. Each interview concluded with a brief socio‐demographic questionnaire.

Interviews were audio‐recorded with consent, transcribed verbatim in Arabic, and translated into Hebrew for collaborative analysis. The accuracy of the translations was assured by back translation and verification by an independent bilingual translator [43]. At a later stage, while preparing this manuscript, participant quotes presented in the Findings section were translated into English using the same method. Translation was treated as an interpretive act rather than a mechanical one, with both authors reviewing key quotations for meaning fidelity. Pseudonyms that were chosen by the participants during the interviews were applied, and identifying details removed. Member checking was not conducted due to participants' health status and the study's single‐session design; however, analytic rigor was reinforced through reflexivity, memoing, and collaborative review.

### 2.4 Data Analysis

Data analysis followed a reflexive thematic analysis approach [44, 45], guided by the context‐informed constructivist framework underpinning the study. Analysis was understood as an active, interpretive, and reflexive process in which themes were generated through sustained engagement with the data rather than discovered as fixed entities. The analysis was performed in Hebrew, thereby allowing both authors to engage iteratively with the transcripts, reading and re‐reading the interviews to attend to patterns of meaning, culturally embedded discourses, and contextual influences shaping participants' accounts.

Initial coding was inductive and interpretive, emphasizing meaning‐making rather than content categorization, and was conducted separately by both authors. Codes were treated as analytic tools that evolved through reflexive engagement with the data, capturing participants' constructions of self, illness, faith, family, and care. Initial theme development, performed by the first author, involved moving from descriptive patterns to interpretive abstraction, with ongoing comparison across interviews to explore convergence, divergence, and contextual nuance. Consistent with reflexive thematic analysis, analytic rigor was not sought through coding consensus or inter‐rater reliability, but through reflexive dialogue between the authors, memoing, and iterative refinement of interpretations, recognizing the researchers' positionalities as integral to the analytic process [45]. The relationships among these themes were developed by both authors through continuous discussion until the thematic map was finalized (see Table 2). The final thematic structure reflects interwoven lived meanings shaped by sociocultural, developmental, and political contexts, and offers a clinically meaningful framework for understanding psychosocial adaptation in young adults with cancer; this framework is illustrated in Figure 1.

**TABLE 2:** Thematic map.

#	Theme	Sub‐theme
1	Inener societal and cultural lived meanings of illness	Use of the explicit term *Cancer* in a social‐cultural context
		Illness concealing and maintaining secrecy in arab society
		The primary role of religion and faith in god
2	External lived meanings of cancer treatment in israeli hospitals	The hospital as a politically neutral safe haven
		Challenges in cross‐cultural interactions within the hospital

**FIGURE 1:**
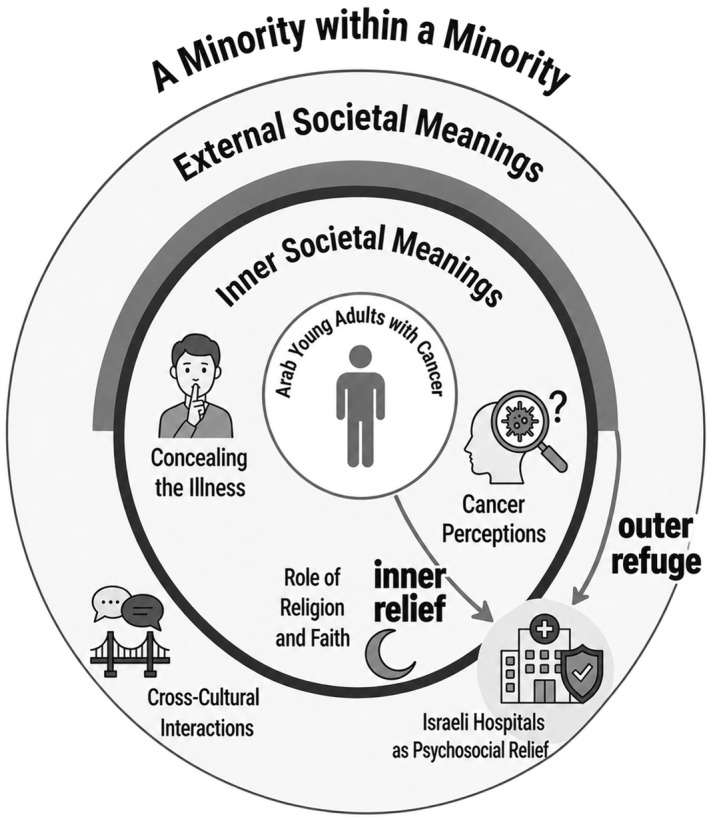
Contextual interactions among the lived meanings of cancer for arab young adults.

### 2.5 Ethical Considerations

Ethical approval was obtained from the Hadassah Medical Center Institutional Review Board and Ethics Committee. All participants provided written informed consent outlining voluntary participation, confidentiality, privacy, anonymity, and data protection. As stated above, recruitment was challenging, resulting in trust issues and fear of exposure. To enhance trust and strengthen participants' sense of protection and anonymity, all participants were asked to choose a pseudonym. The pseudonyms were used during analysis and identifying details omitted. Physicians involved in recruitment were not informed about who agreed to participate or what was disclosed during interviews. Given the small community and sensitivity of illness disclosure, special care was taken to anonymize quotes and mask identifying details.

### 2.6 Trustworthiness and Reflexivity

As detailed below, trustworthiness was enhanced through reflexivity, methodological transparency, and collaborative analysis. The study addressed credibility through prolonged engagement and analytic memoing, dependability through documentation of analytic decisions, confirmability via reflexive journaling, and transferability through rich contextual description.

Shkedi [42] highlights the importance of qualitative researchers maintaining awareness of their initial conceptual perspectives while remaining open to evolving insights throughout the research process. Researchers inevitably carry preconceptions shaped by their professional and personal backgrounds. In context‐informed qualitative research, attentiveness to power dynamics between researchers and participants is crucial, and reflexivity serves as a key strategy for fostering such awareness [46].

The research team comprised two authors bringing complementary positionalities. The second author, an Israeli‐Jewish clinical‐health psychologist, leads a Psycho‐oncology service in a large tertiary hospital; the first, an Arab social worker and graduate student, conducted all interviews in Arabic. Their collaboration embodied both *insider* and *outsider* perspectives [47]. The second author's institutional role offered access and interpretive distance; the first author's shared language and cultural background fostered trust and intimacy but also required reflexive awareness of over‐identification. Their continuous dialogue throughout data collection and interpretation functioned as a form of reflexive triangulation, illuminating how researcher identities inevitably shape meaning‐making. Rather than minimizing these differences, the team treated them as analytic data, mirroring the cross‐cultural encounters participants described. This dual positionality enriched analysis by illuminating the intersections of culture, care, and power that constitute the lived experiences of Arab YA with cancer in Jerusalem.

## 3 Findings

This study explored how Arab young adults from East Jerusalem experienced and made meaning of cancer as members of two intersecting minority social groups, while navigating multiple sociocultural, religious, and national contexts. The analysis revealed two overarching themes, with subthemes reflecting how participants negotiated the (1) inner societal and cultural lived meanings of illness and (2) the lived meanings of receiving care in Israeli hospitals, which were external to their daily lives. Together, these themes illuminate cancer as a deeply contextualized experience—one shaped not only by the disease itself but also by the social, linguistic, familial, and political worlds in which participants live. These lived meanings are organized thematically and presented in Table 2.

### 3.1 “I Don't Want to Live in an Atmosphere of Being Sick”: Inner Societal and Cultural Lived Meanings of Illness

#### 3.1.1 “The Word Itself Weakens You”: Use of the Explicit Term Cancer in a Social‐Cultural Context

Discomfort with the term *cancer* was expressed by 11 of the 15 participants, which they felt diminished their self‐perception and imposed a stigmatizing identity associated with weakness, helplessness, and imminent death. The cultural sensitivity surrounding the term was also evident, as some participants deliberately avoided its use during interviews, instead employing alternative descriptors such as “the disease,” “that condition,” “the tumor,” or “the situation.” Rahma, a 45‐year‐old woman, married with five young children, diagnosed with breast cancer a few months prior to the interview, articulated her reluctance to be identified as a “cancer patient” without explicitly using the term:

I don’t like being called a patient of such‐and‐such, because to people it’s the end of the world. Personally, I accept the illness, but people view me as weak and pitiful. The name itself weakens the person. I don’t want pity because I’m weak. Those around you don’t think like you; they see you as a pity case. Some people start whispering around you as if you’re contagious or powerless.

Rahma's words conveyed how language itself becomes a site of stigma. Her words illustrate her deliberate avoidance of the term cancer as a coping strategy to preserve her sense of strength and dignity within a society that often perceives cancer patients as weak, unfortunate, and terminally ill. By concealing her illness, she maintained a perception of resilience and avoided being viewed as vulnerable, thus distancing herself from associated stigmas, choosing to conceptualize cancer as a manageable condition. Additionally, nine other participants similarly discussed the complexities surrounding the use of the term cancer and its cultural connotations. Avoidance of the term may also reflect deeper concerns, like fear of contagion, hereditary transmission, or diminished social and familial standing.

Positioning herself differently, Julia (34), a mother of three who had been living with breast cancer for 3 years, shared the social and familial challenges she faced post‐diagnosis:

I use the scientific term: “I am a cancer patient.” I have no issue with the name of the disease. Yes, some people react… “God forbid,”… But I didn’t have a problem saying it. […] My mother, for instance, would always tell me not to say it, and I would ask her, “Why not?” It’s not something I should feel guilty about. […] You know, some people are afraid of the word itself, and others worry that it’s contagious, even though it’s not. […] My mother‐in‐law, for example, would say, “Where did you bring this [cancer] from?”.

Julia's account illustrates her awareness of the sociocultural challenges surrounding the term cancer. However, she adopted a more assertive and accepting stance. Her insistence on speaking the word signified both defiance and self‐definition. Her experience also underscores broader societal fears of contagion and avoidance.

Julia's account also gives a more explicit example of a generational and developmental tension around disclosure. Her willingness to use the biomedical term “cancer” contrasted with her mother's instruction not to say the word and with her mother‐in‐law's question, “Where did you bring this from?” In this sense, naming the illness was not only a personal coping strategy but also a way of negotiating between inherited norms of silence and a more open, self‐defining stance toward illness.

Others, like Izz (27), a married father of two, with recently diagnosed lymphoma, adapted his language to protect his listeners:

Honestly, I avoided it, not for myself but for the person I’m speaking to. When I say “cancer,” it’s as if I said something they can’t digest. Anyone you tell, “I have cancer,” you’ll see their face change—they panic and don’t know how to react. Their whole expression shifts […] The illness itself doesn’t bother me; it’s the way people behave that hurts and disturbs me.

For Izz, self‐censorship served compassion; silence shielded others from discomfort even as it isolated him. His cautious approach underscores the tension between personal acceptance and the social consequences of disclosing his diagnosis.

Nearly all participants (11/15) discussed the challenges associated with explicitly using the term “cancer,” though their responses varied. Together, these accounts revealed how naming, or avoiding, cancer carry complex sociocultural connotations. Thus, adding a layer of psychological burden, as participants managed both their health and the symbolic implications of their diagnosis in their social worlds.

#### 3.1.2 “In Our Culture, Someone Diagnosed With This Illness Is Seen as Doomed”: Illness Concealing and Secrecy in Arab Society

Avoidance of the term *cancer* was part of a broader pattern of secrecy, a dominant cultural norm across interviews. In Arab society, rooted in collectivist values, many participants described how disclosure was selectively limited, even among close family members. More than half of the participants chose to keep their diagnosis hidden. Their reasoning centered on avoiding pity, undue attention, and preserving a sense of normalcy. The decision to conceal their diagnosis underscores the tension between individual coping strategies and societal expectations regarding illness and vulnerability. Mariam, a 32‐year‐old mother of two who had to pause her career as a healthcare professional due to breast cancer treatment, shared her perspective:

Since I was diagnosed, I decided not to share and chose to live a normative life or at least try to. […] I imagine sitting in some gathering, and whether I am well or not, they will only focus on that topic [her illness]. I don’t want to live in an atmosphere of being sick. I want to handle the emotional and physical aspects myself and continue my life as usual.

Mariam's narrative illustrates secrecy as an active and protective choice—an effort to shape how others perceive her and to avoid the shift in identity that disclosure would entail.

For young adults, however, secrecy was not only a response to generalized stigma. Participants also linked disclosure and concealment to age‐salient concerns about marriage, childbearing, parenting, family responsibility, and future social positioning. Hanan, age 34, who was married and did not yet have children, described the diagnosis as disrupting the expected sequence of marriage and family formation:

If I had not been married, maybe it would have been easier. If I had received the diagnosis before marriage. Now there is someone I feel is waiting for me, and I feel that I cannot do anything for him. I do not know whether to say thank God that I do not have children, or whether it would have been better if I did have children. I do not know whether I will have children or not.

Hanan's words show how cancer was experienced not only as a threat to health, but also as a disruption of young‐adult relational futures. Marriage and possible motherhood became part of the illness meaning itself, shaping how the diagnosis was understood, feared, and socially positioned.

Similarly, Naya (38), mother of four, opted to withhold her diagnosis, even from her immediate family. Her primary concern was evading pity and resisting societal perceptions that equate cancer with inevitable death. She explained:

I didn’t want them to pity my children, as if they were already finding my replacement. When a person is ill, they need to face it themselves. In our culture, someone diagnosed with this illness is seen as doomed, someone who will receive chemotherapy and then die.

Naya's account underscores her decision to conceal her illness as a means of shielding herself and her children from pity. Her preference reflects a broader mistrust in the community's capacity to offer meaningful support. Instead, she perceived societal reactions as a potential source of distress rather than comfort.

For participants who were already parents, disclosure decisions were also shaped by the wish to protect children from fear and preserve parental stability. Miriam (32) described knowing that treatment would involve fatigue and pain, but framed her main concern around her children rather than herself:

I knew what the consequences of the treatment would be, and my greatest concern was not coping with the fatigue and the pain, because that would pass and I would feel better. I was more worried about how I would cope with my children. I did not want them to feel it, because after all, they are still young.

Miriam's account illustrates how concealment and emotional restraint could function as child‐protective strategies. Rather than reflecting denial, limiting what children sensed or knew allowed participants to continue occupying the role of a responsible parent within the family.

Julia (34) similarly linked illness to guilt over disrupted maternal responsibilities:

At some point I felt guilty… I am not with them much, I do not help them with their homework as I should, I am not present at school events as I should be.

Four participants shared that they reluctantly disclosed their diagnosis which was then followed by overwhelming and unhelpful reactions. Reem (38), mother of five diagnosed with breast cancer, shared her struggle with her family's reaction after her husband disclosed her illness:

It was very, very hard. My husband told the family. As soon as I began the biopsies, everyone already knew. Because we had a prior experience with my father, it was very difficult for everyone. As Arabs, we tend to seize these moments to cry and mourn. At some point, I couldn’t handle it anymore. I didn’t want to see anyone or sit with anyone because I didn’t want them to pity me or cry for me.

For Reem, unsolicited disclosure intensified emotional distress and stripped her of control over her illness narrative.

Overall, participants framed secrecy not as denial or a form of avoidance but as emotional boundary‐setting, a culturally resonant way of preserving self‐respect and shielding loved ones from fear, gossip, or stigma.

#### 3.1.3 “I Feel There's a Direct Connection Between Me and God”: The Central Role of Religion and Faith in God

Religion and faith emerged as salient across the sample, with many participants expressing gratitude for the opportunity to deepen their spiritual connection. More than half of the participants perceived cancer not as a random occurrence but as a divine test of faith and resilience. Faith provided comfort during moments of pain, weakness, loneliness, and uncertainty. Participants described their connection with God as a primary source of support, especially when human support was limited or complicated by secrecy. Maha, a 43‐year‐old mother of three diagnosed with breast cancer, underscored the significance of her relationship with God:

Your only refuge is in praying to Him and seeking His help. Even if you have close people around you, you can’t share everything with them; instead, you prefer to turn to God. I feel there’s a direct connection between me and God.

Zina (40), a breast cancer patient experiencing estrangement from her family, echoed this:

My God is my only support, and He provides me with relief. I feel I’m not alone because God is with me. […] I believe I will recover for my son’s sake.

These accounts demonstrate how faith provided a stable anchor amid uncertainty—a relational and transcendent source of resilience when social networks were strained or inaccessible. For many participants (10/15), faith served as both meaning‐making and emotional containment, enabling participants to navigate fear, isolation, and physical suffering.

### 3.2 “Inside the Hospital There's None of That”: External Lived Meanings of Cancer Treatment in Israeli Hospitals

Beyond the sociocultural experiences within their local Arab society, more than half of the participants also emphasized the broader cultural and national context shaping their experiences. The intersection of cultural, social, and national factors was particularly evident in participants' encounters, often for the first time, with medical professionals in Israeli hospitals. While many described these cross‐cultural interactions positively, four participants reported challenges, including perceived disparities, inequality, and discrimination.

#### 3.2.1 “Once We Arrive at the Hospital, the Care Changes”: The Hospital as a Politically Neutral Safe Haven

Half of the participants highlighted a stark contrast between their experiences at Israeli hospitals compared to the broader public sphere. Within the hospital setting, they reported not encountering racism, discrimination, or exclusion—experiences they often faced in other public spaces. Despite the complex political reality outside, participants expressed a strong preference for receiving treatment at Israeli hospitals, viewing them as neutral spaces where they felt safe and well cared for. Reem (38), residing in a village in the J2 area, which is not under Jerusalem's municipal jurisdiction and therefore requires a special medical pass to travel into Israel, noted:

Even though my husband is willing to fly me anywhere in the world, I chose and preferred to be treated here [an Israeli hospital]. I have to get up at 5:30 a.m. just to make it here by 8:30. Our situation is very complex because of the checkpoint… But once we arrive at the hospital, the care changes and becomes more accessible. […] Especially in the context of illness and treatment, I don’t think there is racism.

For Reem, the hospital stood in opposition to the political tensions surrounding her daily life. This contrast was echoed by others who experienced a similar dissonance between the outside sociopolitical climate and the attentive care provided by the predominantly Jewish‐Israeli healthcare staff. Heba (45), a mother of four who had been undergoing treatment for colon cancer at the same hospital for 8 years, reflected on the distinct atmosphere within the hospital:

Here, they care deeply, are compassionate, and give wholeheartedly. Outside, there is racism, and it’s hard sometimes, but inside the hospital there’s none of that. Even when the situation outside is tense, I have never experienced inappropriate treatment in the hospital.

These accounts portray the hospital as a liminal space—separate from political conflict, experienced as both safe and humanizing.

This perceived neutrality functioned as a sanctuary, offering a reprieve from the socio‐political realities outside the hospital. It also reflects the hospitals' efforts to foster a supportive environment for patients navigating life‐threatening illnesses.

A third of the participants also highlighted the emotional impact of supportive healthcare staff. Conan (23) described the crucial role of a social worker in shaping his experience:

The person I felt influenced me most was a social worker. … I felt he was the best person for me. He would come to meet me, […] He said he’d help me with anything I needed. […] He was amazing.

These narratives underscore how compassionate care can mitigate broader sociopolitical vulnerabilities, reinforcing the hospital's role as a sanctuary.

#### 3.2.2 She Would Dismiss My Response, Saying, “No, you're Not Fine”: Challenges in Cross‐Cultural Interactions Within the Hospital

While many participants perceived the hospital as a politically neutral sanctuary, the hospital was not uniformly experienced as neutral. Four of the participants described cultural misattunements or moments of misunderstanding that disrupted trust.

Some reported feeling misunderstood by hospital staff, particularly regarding culturally sensitive decisions. Mariam (32), mother of two, recounted difficulty with a Jewish social worker who challenged her coping style:

I didn’t feel like I could open up or share my feelings with her. For example, she would ask me how I was doing. If I answered that I was fine or said 'Alhamdulillah' [praise God], she would dismiss my response, saying, 'No, you’re not fine. Say that you’re not okay, express yourself, share your exhaustion.' When she asked if I had told my friends and family and I said I didn’t want to share, she opposed that too.

Here, culturally grounded expressions of endurance, such as *Alhamdulillah,* were misunderstood as denial, highlighting how therapeutic norms can clash across cultural lines, thereby hindering trust in the healthcare staff. In some cases, cultural misunderstandings led to perceptions of discrimination and rejection.

Julia described such feelings after her non‐citizen status was discovered:

I met with a social worker, but once she found out I wasn’t Israeli, she stopped speaking to me or meeting with me. I was surprised. […] I was very surprised, but it passed.

This moment of silence was experienced as discriminatory, adding emotional strain to an already vulnerable situation.

The same participants also reported experiences of neglect, misjudgment, or insufficient cultural understanding from hospital staff. Sara (34), mother of two with lymphoma, reflected on initial neglect that later shifted to attentive care:

They neglected me at first […] they should have done more… more tests, more investigation, to sit with me longer, to really see me and analyze my situation. I may have felt pain that I couldn’t fully express. Later, during my hospital stay, there were problems in Jerusalem, but honestly, I didn’t encounter bad treatment from the medical staff or the clerks. They didn’t make me feel bad—in fact, I felt like I was receiving compensatory care.

Sara's narrative exemplifies the emotional complexity of navigating healthcare as an Arab patient, oscillating between moments of misrecognition and genuine repair. This dynamic has important implications for trust and continuity of care.

Together, these accounts show that while Israeli hospitals often functioned as welcoming spaces, cultural gaps and systemic inequities occasionally surfaced, echoing the broader sociopolitical tensions participants lived with daily.

## 4 Discussion

This study explores the psychosocial experiences of Arab YA coping with cancer while receiving treatment in two Israeli hospitals. Simultaneously positioned within social and biomedical minority groups, these participants offer a distinct perspective on how these YA navigate illness across their sociocultural, political, and institutional environments. Grounded in a context‐informed, constructivist framework, the findings illustrate how cancer is experienced not only as a biomedical event but as a culturally embedded and socially mediated phenomenon. Two interrelated themes emerged: (1) the inner societal and cultural lived meanings of cancer, and (2) the lived experience of receiving care in predominantly Israeli‐Jewish hospitals—an environment external to the YA's daily lives. Together, these lived meanings highlight three analytically distinct but overlapping contexts central to this dual‐minority positioning: (1) the inner sociocultural context, in which stigma, secrecy, and communal expectations shaped cancer as a challenge and rendered the hospital a potential source of psychosocial relief; (2) the context of young age, including evolving perspectives on tradition and modernity; and (3) the external political‐national context, in which the hospital was at times experienced as a relatively protected or relieving space within a broader environment marked by inequality and tension. Understanding these lived meanings within their context enriches and extends existing literature on YA with cancer [48, 49, 50, 51], offering opportunities for context‐informed active care. Importantly, these findings underscore that young adulthood is not merely a demographic descriptor but a critical psychosocial context in which concerns about identity, fertility, partnership, and future profoundly shape responses to cancer and care.

### 4.1 The Inner Sociocultural Context: Cancer as a Challenge

Across interviews, cancer was described as a deeply contextual experience embedded within social, cultural, religious, and political worlds. For YA living in East Jerusalem, an area marked by socioeconomic disadvantage, limited mobility, and non‐citizen status [33], a cancer diagnosis represented both a personal existential crisis and an amplification of daily structural challenges. Within this context, the hospital was significant not only as a site of medical treatment, but also as a space that could temporarily relieve some of the psychosocial pressures associated with cancer in participants' home and community environments.

Participants' reluctance to use the word *cancer* reflects the moral and symbolic weight of the illness in Arab communities. The term was widely associated with incurability, death, and social decline, aligning with research on cancer fatalism [52] in other Arab and Middle Eastern contexts [21]. This culturally embedded fatalism mirrors psychological models suggesting that diminished perceived control can undermine emotional adjustment and health‐promoting behaviors [53].

For some, avoiding the term also served as a protective strategy against stigmatization. Participants feared that naming the illness would invite pity, social distancing, or moral judgment. This echoes the work of Goffman [54] on stigma and more recent findings on how sympathy can inadvertently reinforce marginalization [55]. Additionally, secrecy safeguarded children's marriage prospects or preserved the family's social standing. This aligns with prior research among Arab women in Israel, where concealment is used to maintain dignity and fulfill expected familial roles [4].

In this context, secrecy served a dual psychological purpose: it protected participants' sense of self and offered a culturally resonant way to regulate disclosure, maintain agency, and preserve dignity. For young adults, these dynamics also carried developmental significance, as decisions about disclosure were closely tied to concerns about marriage, parenting, family role expectations, and future social belonging. Although Western clinical practice often discourages nondisclosure [56, 57, 58], participants in this study described secrecy as an active, intentional coping strategy that strengthened, rather than undermined, their resilience. This aligns with recent studies of disclosure practices among AYA cancer survivors identifying both psychological benefits and burdens linked to disclosure decisions, reinforcing the idea that disclosure is a psychosocial strategy shaped by social support, perceived stigma, and identity concerns [59]. Similar patterns have been noted in other sociocultural settings where norms around honor, privacy, and community perception shape approaches to illness [60].

Interestingly, the dynamics of secrecy also shaped the research process itself. Some potential participants declined involvement due to fear of exposure—even when confidentiality was assured. Despite the first author's shared language and cultural background, her connection to the community deterred some individuals. This highlights the psychological impact of anticipated stigma and underscores the importance of culturally safe, trust‐building approaches in both research and clinical contexts.

### 4.2 The Context of Young Age: Evolving Perspectives of Tradition and Modernity

Although traditional norms emphasizing secrecy and stoicism were prominent, participants' narratives also reflected cultural transformation. A subset of participants described disclosure practices that reflected negotiation between inherited norms of silence and more open, self‐defining approaches to illness. This was most visible in accounts in which participants resisted family members' avoidance of the word cancer, selectively managed what others could know, or framed disclosure in relation to marriage, childbearing, and parenting. Thus, the tension was not only cultural but developmental: cancer threatened the social tasks through which young adulthood is recognized, including forming or sustaining a partnership, becoming a parent, caring for children, maintaining work and family roles, and preserving future social belonging.

At the same time, the present findings should be interpreted in light of the developmental composition of the sample. The present sample was weighted toward participants in the later phase of young adulthood: most were in their 30s or early 40s, and many were partnered and had children, suggesting that their accounts may reflect the later phases of young adulthood more than the earlier stage of emerging adulthood. This is important because young adulthood is not a uniform developmental category: individuals in the earlier part of adulthood are more likely to be negotiating educational or vocational entry, emerging independence, and the formation of intimate relationships, whereas those in later young adulthood are more often managing long‐term partnership, parenthood, work, and broader family responsibilities [8, 10, 11].

This coexistence of tradition and change aligns with broader shifts described in Arab societies, where globalization and modernization intersect with collectivist values [61, 62]. Younger participants in particular described navigating a hybrid cultural space, balancing respect for family expectations with a desire for transparency, autonomy, and connection. In this sense, secrecy and concerns about stigma were shaped by developmental stage: they were tied not only to cultural norms of modesty and concealment, but also to age‐salient concerns regarding partnership, parenting, family responsibility, and future social positioning within the community.

Yet the present findings also suggest that these broader cultural negotiations were shaped by developmental stage within the YA period itself. Accordingly, some of the concerns that emerged in the present study, particularly those related to responsibility toward children, preservation of family roles, and the management of illness within established marital, parental, and communal obligations, may be more characteristic of later YA than of the earlier YA period. In this context, the tendency toward secrecy and the wish to avoid stigma were intertwined not only with personal coping, but also with efforts to protect children, preserve family standing within the community, and prevent possible future harm to children's social and marital prospects in more traditional social settings [4, 18, 20]. Thus, themes such as secrecy and stigma were not simply background cultural features, but were experienced through the developmental realities of young adulthood itself. These findings underscore the importance of approaching the YA category as developmentally heterogeneous and of recognizing that cancer may be experienced differently across phases of young adulthood depending on one's family position, relational commitments, and social responsibilities [10, 11].

Faith and religious practices served as central sources of strength across these cultural positions. For some participants, especially those who concealed their diagnosis or lacked family support, belief in God was described as their most reliable companion. Religious coping provided meaning, hope, and emotional regulation [63] and is consistent with literature in Muslim‐majority contexts, where faith anchors resilience in the face of illness [64]. In this study, spiritual connection functioned both as an existential resource and a relational refuge when social networks felt constrained.

### 4.3 The External Political‐National Context: Navigating Cancer Care in Israeli Society

Participants described Israeli hospitals as spaces that differed markedly from the broader sociopolitical climate of Jerusalem. While daily life was shaped by tensions, segregation, and structural inequalities between Arab and Jewish communities, most participants experienced the hospital as a neutral, safe, and compassionate environment. In this sense, the hospital functioned not only as a medical setting, but also as a form of psychosocial refuge, offering temporary relief from both the social pressures surrounding illness within participants' communities and the wider political tensions of everyday life. For many, hospitals offered psychosocial relief on two levels: a respite from community expectations surrounding secrecy (inner societal lived meanings) and an escape from the political stress and discrimination experienced elsewhere (external societal lived meanings). The relationships among these lived meanings are illustrated in Figure 1.

This contrast can be understood through the lens of Conservation of Resources theory [65], where the hospital environment functioned as a resource‐rich zone that buffered distress. It is also consistent with the ethos of medical neutrality, which aspires to deliver equitable care regardless of political identity [66, 67]. Participants' overwhelmingly positive descriptions suggest that, for many, neutrality was effectively enacted.

However, neutrality is not synonymous with cultural competence. Some participants described subtle yet meaningful encounters of cultural misalignment—such as invalidation of religious expressions, discomfort with nondisclosure choices, or perceived differential treatment. These moments echo Keshet and Popper‐Giveon [67] who caution that strict neutrality can obscure structural inequalities and cultural differences, inadvertently hindering genuine inclusion.

Such ruptures hold clinical significance. The therapeutic alliance—a core predictor of psychological outcomes [68], can be compromised when patients feel misunderstood, even in the absence of overt discrimination. These findings highlight the need for care models emphasizing cultural humility, reflective practice, and context‐informed assessment [69], particularly in cross‐cultural oncology settings.

### 4.4 Cultural Context‐Informed Active Care

Participants identified members of the healthcare team as important sources of emotional and psychological support. Several described the hospital staff in explicitly relational terms, emphasizing compassion, care, and responsiveness. As Heba noted, “Here, they care deeply, are compassionate, and give wholeheartedly,” contrasting her experience in the hospital with the racism and difficulty she encountered outside it. Reem similarly emphasized that once patients reached the hospital, “the care changes and becomes more accessible,” and added that, particularly in the context of illness and treatment, she did not experience racism there. Conan also described the central role of a social worker in shaping his experience: “The person I felt influenced me most was a social worker… He said he'd help me with anything I needed… He was amazing.” Together, these accounts suggest that empathy, emotional availability, and practical assistance were experienced as important sources of support that complemented medical treatment. These relational dimensions parallel known predictors of well‐being among chronically ill individuals [70]. Although no studies have directly examined medical teams as resilience factors in cross‐cultural contexts, existing research highlights the importance of cultural competence, humility, and reflective practice in reducing bias and fostering care that moves beyond neutrality toward a more holistic engagement with patients' experiences [69].

Notably, participants also described meaningful relationships with staff even when cultural differences existed. This suggests that contextual attunement, acknowledging patients' cultural, religious, and national positioning, may foster trust despite broader political tensions. At the same time, the data indicate that supportive care was not experienced uniformly. As described earlier, some participants encountered cultural misattunement or felt misunderstood by staff, particularly around norms of emotional expression and disclosure. Taken together, these findings point to the value of integrating culturally attuned communication, flexibility around disclosure norms, and sensitivity to faith‐based coping into routine psychosocial assessment and intervention with YA patients. In addition, they underscore the importance of developing context‐informed training programs that help healthcare professionals recognize how sociocultural norms, stigma, and disclosure practices shape patients' meaning‐making and coping.

### 4.5 Study Limitations and Recommendations for Future Research

This study has several limitations. Convenience and purposive sampling likely attracted participants who were relatively comfortable engaging with the healthcare system, while individuals with greater distrust may have declined participation, limiting the diversity of perspectives. Most participants were in stable physical and emotional condition, restricting insight into experiences of advanced or terminal illness. The focus on East Jerusalem, while essential to this study's aims, may limit transferability to other Arab communities in Israel or abroad. Additionally, the modest sample size (15 participants), for reasons explained above, precludes subgroup comparisons. Lastly, although the study focused on young adults, the sample was weighted toward the later phase of young adulthood, with many participants in their 30s or early 40s and several occupying established marital and parental roles. Accordingly, the findings may reflect the concerns of later young adulthood more strongly than those of earlier emerging adulthood and should be interpreted with this developmental composition in mind.

Future research should expand to Arab YA receiving oncology care in different regions of Israel and internationally. Comparative studies across sociopolitical contexts could illuminate how minority status shapes illness experiences. Research examining the experiences of spouses, parents, and siblings may also elucidate the relational dimensions of coping within collectivist cultures or minorities.

### 4.6 Clinical and Theoretical Contributions

This study offers a context‐rich understanding of how Arab young adults make meaning of cancer while receiving care in Israeli hospitals. By centering participants' own narratives—shared in their native language and situated within their sociocultural, political, and institutional contexts—the study illuminates lived experiences that have been largely absent from the literature. It brings forward the nuanced challenges faced by this dual‐minority population and deepens awareness of how cultural norms, community expectations, and national structures shape the illness experience. Rather than reflecting competing explanatory frames, these multiple contexts together clarify how illness meaning‐making was produced at the intersection of developmental stage, family and community life, institutional encounters, and broader political conditions.

Beyond its empirical contribution, the study also offers a clearer theoretical contribution. First, the findings extend a context‐informed understanding of illness by showing that cancer is experienced not only as an individual biomedical event, but also as a socially and institutionally mediated experience shaped by intersecting developmental, cultural, and institutional contexts [39]. In this sense, the study supports constructivist approaches that view meaning‐making as embedded within broader social worlds rather than located solely within the individual. Second, the findings deepen existing theoretical understandings of illness meaning‐making by showing that participants' experiences of cancer were shaped not only by the disease itself, but also by interconnected concerns related to secrecy, family roles, faith, social expectations, and community positioning, thereby extending broader conceptualizations of illness as a socially mediated process [54]. Third, the findings suggest that coping and help‐seeking among young adults with cancer are shaped not only by psychological appraisal, but also by minority positioning, linguistic barriers, and the institutional character of care, thus broadening dominant psychosocial models that often emphasize intrapersonal adaptation over structural and relational context [53]. Taken together, the study advances psycho‐oncology theory by showing that the experience of cancer among young adults from minority backgrounds is best understood through a contextual and relational lens that integrates developmental stage, culture, social meaning, and institutional power.

Clinically, the study provides actionable insight for multidisciplinary teams in Israel and in other healthcare settings serving Arab YA patients, especially in minority populations. The findings highlight the need for culturally responsive, context‐informed approaches to psychosocial care—approaches that recognize the roles of religion, family, cultural norms, and political realities in shaping coping and help‐seeking. Disseminating these insights among clinicians, policymakers, and community stakeholders may support the development of more inclusive, attuned, and equitable care for Arab YA navigating cancer.

## 5 Conclusions

This study highlights how cancer among Arab young adults is experienced not only as a medical condition but as a profoundly contextual process shaped by developmental stage, cultural norms, and political‐institutional environments. The findings underscore the importance of attending to young adults' future‐oriented concerns, culturally patterned practices of disclosure and secrecy, and faith‐based meaning‐making, alongside their experiences within cross‐cultural healthcare systems. By foregrounding the voices of Arab YA from East Jerusalem, this study contributes to a more nuanced understanding of how minority positioning intersects with cancer during young adulthood. Clinically, the findings call for context‐informed, culturally responsive psycho‐oncology care that recognizes both the vulnerabilities and the adaptive strategies of young patients navigating serious illness within complex social worlds.

## Author Contributions

Study initiation, primary investigator role, and preparation of the manuscript for publication were performed by Yaffa N. Stokar. Data collection and initial analysis were performed by Rola Abd Elgani. Both authors were engaged in advanced data analysis, theme development and formulation of findings. Initially the study was published as a master's thesis written by Rola Abd Elgani with Yaffa N. Stokar acting as advisor. Parts of the thesis were revised and prepared for publication in the format of the present manuscript, by Yaffa N. Stokar, with assistance from Rola Abd Elgani.

## Funding

The authors have nothing to report.

## Ethics Statement

The study received approval from the Hadassah Medical Center Institutional Review Board and Ethics Committee. All participants gave written informed consent.

## Conflicts of Interest

The authors declare no conflicts of interest.

## Data Availability

The data supporting the findings of this study are available on request from the corresponding author Yaffa N. Stokar.
